# Association between seizure reduction during ketogenic diet treatment of epilepsy and changes in circulatory metabolites and gut microbiota composition

**DOI:** 10.1016/j.ebiom.2024.105400

**Published:** 2024-11-04

**Authors:** Maria Dahlin, Craig Edward Wheelock, Stefanie Prast-Nielsen

**Affiliations:** a Neuropediatric Department, Astrid Lindgren Children's Hospital, Karolinska University Hospital, Stockholm, Sweden; b Department of Women's and Children's Health, Karolinska Institutet, Stockholm, Sweden; c Unit of Integrative Metabolomics, Institute of Environmental Medicine, Karolinska Institutet, 171 77, Stockholm, Sweden; d Department of Respiratory Medicine and Allergy, Karolinska University Hospital, 171 77, Stockholm, Sweden; e Centre for Translational Microbiome Research (CTMR), Department of Microbiology, Tumor and Cell Biology, Karolinska Institutet, 171 77, Stockholm, Sweden; f Department of Clinical Neuroscience, Karolinska Institutet, Stockholm, Sweden

**Keywords:** Gut microbiome, Metabolomics, Pharmaco-resistant epilepsy, Ketogenic diet, Plasmalogens

## Abstract

**Background:**

The ketogenic diet (KD) is a high fat, sufficient protein, and low carbohydrate dietary therapy for drug-resistant epilepsy. The underlying mechanisms of action of the KD remain unclear. In mice, the microbiota is necessary for the anti-seizure effect and specific microbes influence circulatory levels of metabolites that are linked to seizure reduction. However, it remains unclear which changes are linked to seizure reduction in patients with epilepsy.

**Methods:**

We analysed the serum metabolome of children with drug-resistant epilepsy (n = 14) before and after three months on KD. Metabolomic changes were correlated to the gut microbiome and treatment outcome, i.e., seizure reduction.

**Findings:**

In this prospective observational study, we uncovered associations between microbial species and serum metabolites that correlated with seizure reduction. Plasmalogens were most strongly linked to seizure reduction and had significant positive correlations with several gut microbes (*e.g., Faecalibacterium prausnitzii*, *Alistipes communis*, *Alistipes shahii*, and *Christensenella minuta*) while significant negative correlations were found for five strains of *Escherichia coli*. Infant-type Bifidobacteria correlated negatively with other metabolites associated with seizure reduction.

**Interpretation:**

The microbes and metabolites identified here may contribute to the therapeutic effect of the KD in children with drug-resistant epilepsy. Several of these metabolites (*e.g.,* plasmalogens) play important roles in neurobiology and may influence seizures. Based on our findings, anti-seizure therapeutic strategies could be developed involving the targeted manipulation of the gut microbiota and/or its metabolites.

**Funding:**

This study was supported by the Swedish Brain Foundation, Margarethahemmet Society, Sunnerdahls Handikappfond, Stockholm County Council Research Funds, and Linnea & Josef Carlssons Foundation.

**Research in context**

**Evidence before this study**

Epilepsy is ranked as the second most burdensome neurologic disorder worldwide in terms of disability-adjusted life years. One third of epilepsies are refractory to pharmacological treatment, despite a large number of anti-seizure medications available. The ketogenic diet (KD) is an effective alternative treatment of drug-resistant epilepsy. The intestinal microbiota is necessary for the anti-seizure effect of the KD in epilepsy mouse models. Faecal transfers from children on the KD reduce seizures in these mice compared to faecal transfers from the same children collected before starting KD. Mechanisms of how the microbiota may decrease seizures during KD are proposed for these animal models but remain unclear in patients.

**Added value of this study**

We analysed the faecal microbiota and circulatory metabolite profiles of children with drug-resistant epilepsy. Samples were collected prior to starting KD therapy and after three months on the diet. The ketogenic diet induced a metabolic shift in the serum of these children. Notably, seizure reduction during KD was linked to specific gut bacteria and serum metabolites, including the increase of several plasmalogens.

**Implications of all the available evidence**

Plasmalogens have antioxidant properties and inhibit neuroinflammation. Genetic disorders disrupting peroxisome function (*e.g.,* Rhizomelic chondrodysplasia punctata (RCDP) and Zellweger spectrum disorder) are accompanied by plasmalogen deficiency and seizures. However, the association of plasmalogens to specific gut microbes and, importantly, seizure reduction during KD in patients with epilepsy has, to our knowledge, not been reported previously. Our findings may spur the development of anti-seizure therapeutics based on gut microbes and/or their metabolites.

## Introduction

Epilepsy is a common neurological disorder affecting over 50 million people worldwide. Nearly one third of patients with epilepsy do not obtain seizure freedom despite the development of many new antiseizure medications (ASMs).^1^ Pharmaco-resistant epilepsy is defined as failure to achieve seizure freedom following adequate trials of two tolerated and appropriately chosen ASMs.^2^ In children, the ketogenic diet (KD) is a well-established treatment^3^ for pharmaco-resistant epilepsy. The KD is a high fat, sufficient protein, and low carbohydrate dietary therapy. About half of the children on the diet achieve seizure reduction of ≥50%, and 10% achieve seizure freedom.^4^

The KD produces numerous systemic changes in circulatory metabolites including high levels of ketones and lowered levels of glucose. Several hypotheses have been discussed concerning the mechanisms behind the anti-seizure effect of KD, including activation of ATP-sensitive potassium channels, increased inhibitory neurotransmitter release in the brain, such as gamma-aminobutyric acid (GABA) and adenosine, reduced oxidative stress, and enhancement of mitochondrial function.^5^ It is not fully understood how these changes relate to efficacy against seizures. A study on children comparing metabolites in the cerebrospinal fluid (CSF) before and during KD showed significant changes in many lipids and carbohydrates with a stronger response in those that obtained seizure reduction.^6^ Few clinical studies have investigated serum metabolomic changes during KD^7^, ^8^, ^9^ of which two involved patients with epilepsy.^8^^,^^9^

We here applied ‘–omics’ analyses to investigate changes in the gut microbiota, the microbial community residing in the gastrointestinal tract, and serum metabolites related to seizure reduction. The gut microbiota has been shown to influence brain development and neurological function^10^^,^^11^ and was indispensable for the anti-seizure effect of the KD in two epilepsy mouse models.^12^ We have previously shown that KD treatment changed both the composition and function of the gut microbiota^13^ and changes in specific bacterial species were associated with seizure response and anti-inflammation.^14^

In this prospective observational study of pediatric patients with pharmaco-resistant epilepsy, we uncover links between seizure reduction, changes in the gut microbiota and serum metabolites during KD treatment. These bacteria and metabolites may thus play an important role for the therapeutic effect of the ketogenic diet in children with pharmaco-resistant epilepsy.

## Methods

### Patients

The study was conducted at the Neuropediatric Department, Astrid Lindgren Children's Hospital, Karolinska Hospital. All patients had a diagnosis of epilepsy and attended the Epilepsy Outpatient Clinic. Due to resistance to anti-seizure medications, a KD was implemented. The inclusion criteria were: age 2–17 years; pharmaco-resistant epilepsy, no medical contraindications to treatment with KD, and informed consent for participation in the study. Exclusion criteria were antibiotics or probiotics taken within three months before the sample collection. Between five and eight patients starting KD treatment per year met these criteria. In total 14 patients participated. Demographic information including sex and age was retrieved from the medical journals.

### Study design

The efficacy of the KD was determined by response to the diet concerning seizure frequency. The seizure response was calculated from seizure calendars. Before diet initiation, the parents or other caregivers were given seizure calendars used routinely in our clinic and were instructed to make daily notes on the number and types of seizures of their child. The mean seizure frequency the month before initiation of the KD was considered as baseline and was compared with the mean frequency the month preceding the follow-up visit at three months on diet. The mean relative seizure reduction was used as outcome measure. Children with ≥50% seizure reduction were also classified as responders and those with <50% reduction as non-responders. We followed a standardised protocol for the classic KD, which is a modified version of the protocol of the Johns Hopkins Hospital,^15^ further described in.^13^

All meals were calculated for the individual child by a specialised dietician. The ratio of the meal content of fats to proteins and carbohydrates was increased stepwise starting at 2:1, and the optimal ratio for the individual child was usually reached within 3–6 weeks. This ratio was kept unchanged until the follow-up visit at three months after diet start when KD was evaluated concerning efficacy. The children were supplemented with multivitamins and minerals, including potassium, calcium, magnesium, zinc, selenium, and were also provided with 100 mg/kg/day of carnitine. When possible, children continued with unchanged ASMs in type and dose from start to follow-up of KD at three months to minimise confounding factors.

At initiation of KD and after three months on KD, levels of blood glucose and beta-hydroxybutyrate (β-OHB) were measured (Table 1). Venous blood samples for metabolomics analyses and faecal samples for analyses of the gut microbiome were also collected.

**Table 1:** The patients participating in the study (n = 14).

Pat no	Sex	Age at KD start (yr)	Age at seizure onset (yr)	Epilepsy type	Seizure type(s)	Etiology	ASMs prior to KD (no)	ASMs during KD	Comorbidity	KD at 3 months		Outcome	
										Ratio	β-OHB (mmol/L)	Respons	% seizure reduction
1	F	15.3	0.1	F	FIA, GTC	genetic	4	LTG	ID, Mot	3	1.4	Resp	100
2	M	14.8	6.0	F	FIA, GTC	structural	8	VPA	ID, Mot	3.5	1	Resp	99
3	M	17.8	12.7	G	GTC, Ab	unkown	5	VPA, LTG, LCM, CLB	0	3.5	0.8	Resp	93
4	F	11.0	0.1	G	Myocl, Aton, aAb	unkown	11	LTG, ZNS, LCM, CLB	ID, Mot	3	5.7	Resp	85
5	M	2.8	0.4	G	Myocl, Aton	genetic	5	VPA, TPM, VGB	ID, Mot	3.5	5.6	Resp	70
6	F	7.9	0.2	G	Ton	genetic	5	ZNS, LCM, CLB	ID, Mot	3.5	5.2	Resp	62
7	F	5.0	2.3	G	Ton	genetic	8	TPM, CLB	ID	4	5.2	Resp	53
8	M	6.8	0.6	G	GTC, Myocl, Aton, aAb	genetic	6	TPM,ETM, LEV,CLB	ID	3	6.6	Nonresp	40
9	F	14.2	0.6	F	FIA, GTC	genetic	6	CBZ, LCM	ID	3.5	4.9	Nonresp	11
10	F	7.9	5.8	F	Ton, FIA	structural	4	LCM	ID, Mot	4	3.8	Nonresp	10
11	F	8.3	0.3	G	Ton, FIA	genetic	8	LEV, CLB	ID, Mot	4	6.1	Nonresp	10
12	F	3.4	0.1	G	GTK	genetic	9	VPA, CZP	ID, Mot	4	5.7	Nonresp	10
13	M	8.2	0.8	F	ESpasms, FIA	genetic	10	VPA, TPM, LCM	ID	4	4.6	Nonresp	0
14	F	7.6	4.1	G	GTC	genetic	3	VPA	0	3	4	Nonresp	0

Abbreviations: F, female; M, male. yr, years. F, focal; G, generalized. FIA, focal impaired awareness seizures; GTC, generalized tonic clonic seizures; Myocl, myoclonic seizures; Aton, atonic seizures; ESpasms, epileptic spasms; aAb, atypical absence seizures; Ton, tonic seizures. ASMs, Antiseizure medications. LTG, lamotrigine; CBZ, carbamazepine; LCM, lacosamide; VPA, valproate; TPM, topiramate; ETM, ethosuximide; LEV, levetiracetam; CLB, clobazam; ZNS, zonisamide; CZP, clonazepam.

ID, Intellectual disability; Mot, motor dysfunction. β-OHB, beta-hydroxybutyrate. Resp, responder; Nonresp, Nonresponder.

### Sample collection, transport & storage

Blood samples were collected at our Epilepsy Outpatient Clinic by trained nurses and were obtained at the same time as other routine blood laboratory tests for KD treatment. All blood samples were taken in the morning before breakfast and intake of the regular dose of ASMs. The first blood sample was obtained within a week before starting KD treatment and the second after three months on KD at time of follow-up visit. The samples were collected in standard serum tubes without anticoagulant and cold-centrifuged for 30 min at 1400×*g*. The serum was aliquoted into Eppendorf tubes, immediately frozen and stored at −70 °C. All samples were analysed for metabolomics within the same time frame.

Faecal samples were collected by the parents using a sterile FLOQSwab™ (Copan). The first sample was taken during the day before starting KD or in the morning of the day of starting. The second sampling at three months on KD was collected at home and the swabs were kept in a refrigerator for a few hours until transported to the hospital on ice and immediately stored at −70 °C.

### Ethics

The present study was approved by the Ethics Committee of the Karolinska Hospital (“Regionala etikprövningsnämden i Stockholm”, Dnr 2014-117732, 2019-00744 and 2020-03843). Informed consent was obtained from the legal guardians of the children and, if possible, also from the children. This research was conducted in accordance with all relevant guidelines and procedures.

### Serum sample processing

#### Untargeted mass spectrometry analysis

Serum samples were analysed using untargeted metabolomics standard procedures at Metabolon, Inc. (Durham, North Carolina). The following controls were included: a pooled matrix sample generated by taking a small volume of each experimental sample which served as a technical replicate throughout the data set; extracted water samples served as process blanks; and a cocktail of QC standards that were carefully chosen not to interfere with the measurement of endogenous compounds were spiked into every analysed sample, allowed instrument performance monitoring and aided chromatographic alignment. Experimental samples were randomised across the platform run with QC samples spaced evenly among the injections.

Four aliquots of each sample were analysed as follows: 1) using acidic positive ion conditions following elution from a C18 column ((Waters UPLC BEH C18-2.1 × 100 mm, 1.7 μm) chromatographically optimised for more hydrophilic compounds, 2) using acidic positive ion conditions optimised for more hydrophobic compounds. 3) using basic negative ion optimised conditions using a separate dedicated C18 column. 4) via negative ionisation following elution from a HILIC column (Waters UPLC BEH Amide 2.1 × 150 mm, 1.7 μm). The MS analysis alternated between MS and data-dependent MSn scans using dynamic exclusion. The scan range varied slightly between methods but covered 70–1000 *m*/*z*.

#### Identification of metabolites

Metabolites were identified through automated comparison of the ion features in the experimental samples with a reference library of chemical standard entries that included retention time, molecular weight [*m*/*z*], preferred adducts, in-source fragments, and associated MS spectra. Metabolons proprietary visualisation and interpretation software was used to confirm the consistency of peak identification among the various samples. Library matches for each compound were checked for each sample and corrected if necessary.

#### Metabolite Quantification and Data Normalisation

Peaks were quantified using the area under the curve. The raw area counts for each metabolite in each sample were normalised, using the median value for each run day to correct for variation due to instrument inter-day tuning differences. The medians were set to 1.0 for each run and missing values were imputed using the observed minimum after normalisation.

#### Classification of metabolites and Pathway Enrichment analyses

Quality-controlled data were structured into curated metabolic classes and pathways based on the integration of literature and institutional knowledge. For each individual pair-wise comparison, Pathway Enrichment displays the number of statistically significantly different compounds relative to all detected compounds in a sub-pathway, compared to the total number of statistically significantly different compounds relative to all detected compounds in the study. A pathway enrichment value greater than one indicates that the pathway contains more significantly changed compounds relative to the study overall, suggesting that the pathway may be a target of interest for further investigation. The enrichment value for a sub pathway is independent of the directionality (increased or decreased) of the significantly altered metabolite in a particular sub pathway. Pathway Enrichment was based on the hypergeometric distribution, and p-value calculations were performed in R 4.2.1 (http://cran.r-project.org/) using the built-in stat package which have functions for the Hypergeometric distribution.

#### Quantitation of Short Chain Fatty Acids (C2 to C6)

Serum samples were analysed for eight short chain fatty acids: acetic acid (C2), propionic acid (C3), isobutyric acid (C4), butyric acid (C4), 2-methyl-butyric acid (C5), isovaleric acid (C5), valeric acid (C5) and caproic acid (hexanoic acid, C6) by LC-MS/MS (Metabolon Method TAM148). The samples were spiked with stable labelled internal standards and homogenised and subjected to protein precipitation with an organic solvent. After centrifugation, an aliquot of the supernatant was derivatised. The reaction mixture was injected onto an Agilent 1290/AB Sciex QTrap 5500 LC MS/MS system equipped with a C18 reversed phase UHPLC column coupled to a mass spectrometer operating in negative mode using electrospray ionisation (ESI). Quantitation was performed using the peak area of the individual analyte product ions and weighted linear least squares regression analysis generated from fortified calibration standards prepared immediately prior to each run. LC-MS/MS raw data were processed using AB SCIEX software Analyst 1.6.3.

Three levels of QC were prepared in serum by diluting with PBS and/or spiking with stock solutions to obtain the appropriate concentrations for each level. Sample analysis was carried out in a 96-well plate format containing two calibration curves and six QC samples (per plate) to monitor assay performance. Accuracy was evaluated using the corresponding QC replicates in the sample runs. QCs met acceptance criteria at all levels for all analytes.

### Faecal metagenomics analysis

The whole metagenomics-sequenced faecal samples have been previously described.^14^ The taxonomic profiles used here are published as Supplementary Table S1 in.^14^

### Data analysis

All data analysis and visualisation were performed in R version 4.2.2 (2022-10-31) using the following packages: vegan 2.6–4, EnhancedVolcano 1.12.0, mdatools 0.13.0, mixOmics 6.19.4, ggplot2 3.3.6., and pheatmap 1.0.12.

Permutational Multivariate Analysis of Variance (PERMANOVA) and Non-metric MultiDimensional Scaling (NMDS) was performed in the vegan package using Euclidean distances of centred log ratio transformed abundances. We calculated Adonis R2 using the adonis2 function and 9999 permutations.

For multivariate analyses, data values were autoscaled using mdatools and multilevel principal component analysis (PCA) and sparse partial least squares discriminant analysis (sPLS-DA) were performed on the untargeted metabolomics data. For the multilevel parameter, patientID was used to take repeated measures, i.e., pre- and post KD, into account. PCA was run for two components using the pca function in mixomics and default settings i.e., center = TRUE, scale = FALSE, and patientID as the multilevel parameter. For sPLS-DA the following parameters were used: ncomp = 3, validation = “loo”, dist = ‘mahalanobis.dist’, scale = TRUE, auc = TRUE, near.zero.var = TRUE, multilevel = meta$PatientID, thus applying leave-one-out cross-validation, scaling, adjustment for sparse data (nearZeroVar). The number of components (3) and use of mahalanobis distance was based on first running plsda and perf function in mixomics to compute evaluation criteria for PLS-DA where mahalanobis.dist was shown to perform best using the perf function. Highest accuracy of the model (100%) was achieved using five features in one component. A confusion matrix was created based on classification predictions of the final sPLS-DA model.

### Statistics

For the statistical comparisons in the cohort description, Mann–Whitney U test was applied in Statistica (version 8).

A paired t-test was used for analysis of metabolite changes from time point 1 (before KD) to time point 2 (three months after starting KD), Benjamini-Hochberg false discovery rate (fdr) was calculated as well as fold changed (TP2/TP1). Pathway Enrichment Analysis was based on the hypergeometric distribution, and p-value calculations were performed in R 4.2.1 using the built-in stat package. All other statistical analyses were performed in R version 4.2.2 (2022-10-31).

To analyse correlations between metabolites or microbial species to relative seizure reduction, relative changes from time point one (before starting KD) to time point two (three months after starting KD) were calculated as follows:

For metabolites:

fold change(metabolite_(*1 … n*)_) = metabolite_(*1 … n*)*timepoint2*_/metabolite_(*1 … n*)*timepoint1*_

For microbial species:

deltaCLR(taxa_*1 … n*_) = CLR(taxa_1 … *n*_)_*timepoint2*_ - CLR(taxa_1_ … _*n*_)_*timepoint1*_

Spearman's rank correlation coefficient, bias-corrected accelerated bootstraped 95% confidence intervals (CIs), p-value, and false discovery rate (fdr) were calculated between changes of metabolites or species and relative seizure reduction, as well as for metabolites vs. species.

### Data availability

Raw sequencing data are available at the European Nucleotide Archive under study accession number PRJEB28847 and PRJEB49521. Normalised metabolomics data for each sample and each metabolite can be found in Supplementary Table S1.

### Role of funders

This study was supported by the Swedish Brain Foundation, Margarethahemmet Society, Stiftelsen Sunnerdahls Handikappfond, Stockholm County Council Research Funds, and Linnea & Josef Carlssons Foundation. The funders had no role in study design, data collection, analysis, interpretation of data, in the writing of the report; or in the decision to submit the paper for publication.

## Results

### Cohort characteristics

The cohort characteristics are summarised in Table 1. The study cohort consisted of 14 children, nine girls and five boys. The age at KD start was median 8.0 years (Q1 6.8; Q3 14.2). The age of epilepsy onset was median 0.6 years (Q1 0.2; Q3 4.0) with 10 out of 14 having an onset before the age of one year. The classifications of aetiologies, epilepsy types and seizures type were made according to the ILAE classification from 2017.^16^^,^^17^ Most of the children had multiple seizure types and the median number per patient was 2.0 (Q1 1.0; Q3 2.0). The most common seizure types were generalised tonic-clonic seizures and focal seizures with impaired awareness. The children had previously tried a median of 6.0 ASMs (Q1 5.0; Q3 8.0) before diet start. The number of concomitant ASMs at diet initiation were varied between 1 and 4 and the median was 2.0 (Q1 1.0; Q3 3.0). The most commonly used ASMs were valproic acid and clobazam. The rationale was to try to avoid dose changes of AMSs until KD efficacy evaluation at three months, but due to adverse effects, dose reductions of topiramate in two children (22% in patient 13 and 25% in patient 7) and reduction of valproate in one child (37% in patient 2) were necessary. The mean KD ratio at three months on the diet was 3.5 (±SD 0.4) and varied between 3:1 and 4:1. At the timepoint of three months on KD, seven children (50%) were responders with a seizure reduction of ≥50% and seven were non-responders. The percentage of seizure reduction for all participants is given in Table 1. The median level of β-OHB in seizure responders was 5.2 mmol/L (Q1 1.0; Q3 5.6) and in non-responders 4.9 mmol/L (Q1 4.0; Q3 6.1) which was a nonsignificant difference (Mann–Whitney U test).

### The ketogenic diet induces profound changes in serum metabolic profiles

**Fig. 1:**
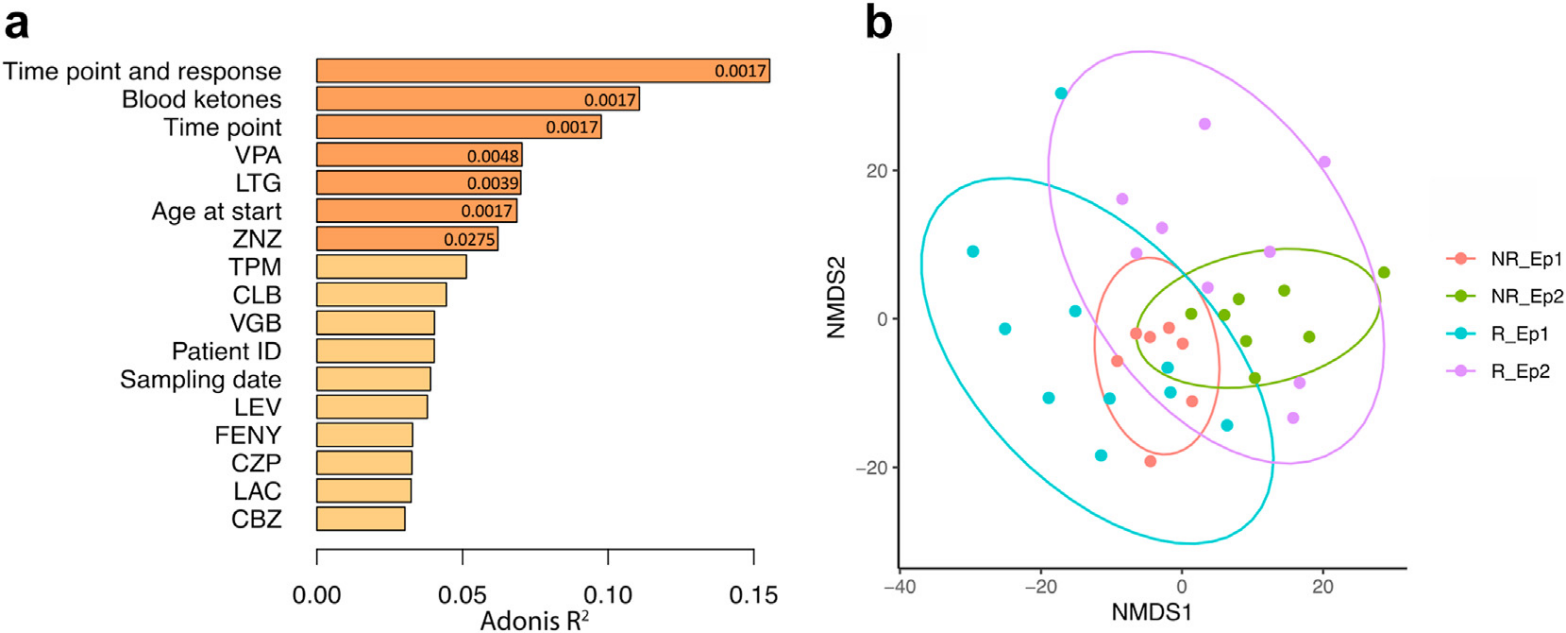
**Serum metabolomic profiles of patients with epilepsy are influenced by treatment and response.** (a) PERMANOVA analysis of covariates influencing the serum metabolomic profiles of the patients ranked by impact (R^2^ value). Significant influence is shown in orange (fdr<0.05), non-significant in yellow. Fdr values below 0.05 are shown inside the bars. (b) NMDS analysis of serum metabolomic profiles with samples from non-responders before KD in red (NR_Ep1), during KD in green (NR_Ep2), responders before KD in turquoise (R_Ep1), responders during KD in purple (R_Ep2).

Analysis of variance in the metabolomic profiles of children with therapy-resistant epilepsy showed that the significant factors with the largest R^2^-value, i.e., the largest variance explained, were related to the dietary treatment (time point and blood ketones) with further increased variance explained, when the patients were grouped into responders and non-responders before and after treatment (Fig. 1a). Age at start and several ASMs (i.e., valproate (VPA), lamotrigine (LTG) and zonisamide (ZNZ)) also influenced the serum metabolomic profiles of our cohort; however, with smaller variance explained than dietary treatment. Remaining ASMs, patient ID and sampling date did not have significant effects on the profiles. Non-metric MultiDimensional Scaling (NMDS) clearly shows shifts across the first dimension (x-axis) due to the dietary treatment, whereas the second dimension (y-axis) showed differences in variance between responders and non-responders (Fig. 1b).

**Fig. 2:**
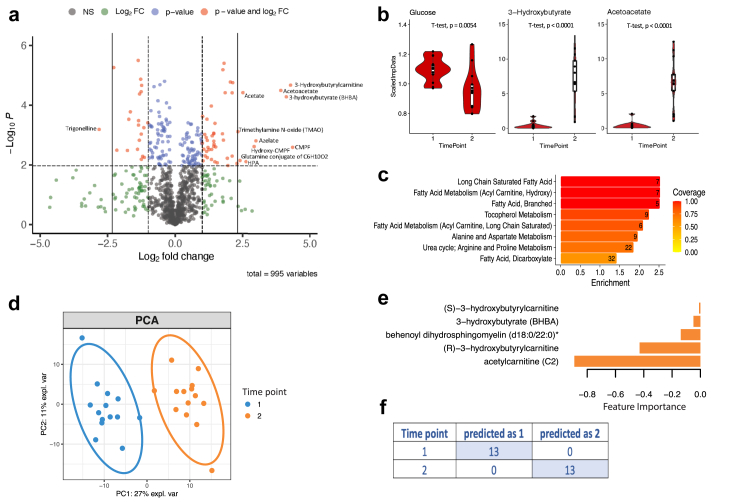
**The ketogenic diet induces metabolomic changes in the serum of children with severe epilepsy.** 345 out of 995 metabolites were significantly changed in a paired t-test (p<0.05) of which 185 remained after correcting for false discovery rate (fdr<0.05) (a) A volcano plot showing significant changes in metabolites due to starting KD treatment, with metabolites significantly changed (p<0.01 and fdr<0.05) above the dashed horizontal line in blue. Metabolites in red showed largest significant fold-changes (FC) with FC>2 beyond the dashed vertical lines and FC>5 beyond the straight vertical lines, labelled with metabolite names. Green indicates metabolite changes with FC>2 but p>0.01 or fdr>0.05. (b) Violin plots of normalised levels of glucose and the major ketone bodies 3-Hydroxybutyrate and Acetoacetate measured in the untargeted assay at both time points. A center line within each box represents the median; box limits are upper and lower quartiles; whiskers show 1.5 × interquartile range; and points depict outliers. (c) Pathway enrichment analysis of significantly changed metabolites with Enrichment scores on the x-axis and bars color-coded by coverage. (d) Multilevel PCA showing unsupervised clustering of serum metabolite profiles by time point. (e) Ranked loading plot of the five metabolites included in the final multilevel sPLS-DA model discriminating samples between time points with 100% accuracy as demonstrated in the confusion matrix in f.

A total of 995 metabolites were detected (Supplementary Table S1), of which 345 significantly changed (p < 0.05, paired t-test) due to KD treatment, 185 after correcting for false discovery rate (fdr) (Supplementary Table S2). The changes in individual metabolites due to starting dietary treatment are shown in Fig. 2a. Metabolites that increased >5-fold during KD include, as expected, the ketone bodies 3-hydroxybutyrate (β-OHB) and acetoacetate as well as the derivative 3-hydroxybutyrylcarnitine. In addition, acetate, trimethylamine N-oxide (TMAO), CMPF and hydroxy-CMPF, azelate and heneicosapentaenoic acid (HPA) increased, whereas trigonelline decreased by >5-fold. As expected, glucose significantly diminished during KD (Fig. 2b) because carbohydrate intake was largely restricted. This was confirmed by blood glucose testing in which a mean level of 4.93 ± 0.45; median 4.8 mmol/L before diet and 4.25 ± 0.46; median 4.1 mmol/L at three months on diet (p = 0.008) was found. The high intake of fat leads to a metabolic shift and production of ketone bodies which were detected here (Fig. 2a and b) and confirmed in blood by quantitative testing for β-OHB (mean 4.3 mmol/L ±SD 1.9; median 5.1; min–max 0.8–6.6; Q1 3.8, Q3 5.7) after three months on KD.

We performed pathway enrichment analysis of significantly changed metabolites to summarise the extensive metabolic changes during KD. Eight pathways were significantly changed during dietary treatment (Fig. 2c) of which five belonged to pathways involving various types of fatty acids. Next, we performed an unsupervised, multilevel Principal Component Analysis (PCA) taking repeated sampling into account. We observed clustering along the x-axis (PC1) of samples from before starting the KD (Time point 1) and after three months on KD (Time point 2) with PC1 explaining 27% of the variation (Fig. 2d). Multilevel Partial Least-Squares Discriminant Analysis (PLS-DA) identified five out of the 995 metabolites as necessary to build a classification model with an error rate of 0% (Fig. 2e and f) demonstrating strong classification properties of these metabolites namely acetylcarnitine, behenoyl-dihydrosphingomyelin (d18:0/22:0)∗, the ketone body 3-hydroxybutyrate (β-OHB) and its derivatives the diastereomers (*S*) and (*R*)-3-hydroxybutyrylcarnitine.

### Specific serum metabolite changes correlate with seizure reduction and changes in the gut microbiota

Next, we analysed whether changes in specific serum metabolites and faecal microbes during KD correlated with treatment outcome calculated as percent seizure reduction. We identify metabolite changes during KD with both positive and negative correlations to seizure reduction of which the top metabolites are listed in Table 2 (see Supplementary Tables S3 and S4 for complete lists). The majority of metabolites are lipids, but carbohydrates, amino acids and peptides were also significant. The strongest positive correlations of seizure reduction were with the plasmalogen 1-(1-enyl-palmitoyl)-2-linoleoyl-GPC (PC P-16:0/18:2) and propionate. Interestingly, while three other, structurally related plasmalogens, namely 1-(1-enyl-palmitoyl)-2-oleoyl-GPC, 1-(1-enyl-palmitoyl)-2-arachidonoyl-GPC and 1-(1-enyl-palmitoyl)-2-arachidonoyl-GPE showed significant positive correlations individually, the strength, significance and confidence of the correlation was largely augmented when the sum of these three changes was analysed, suggesting that a change in any of these three plasmalogens may influence seizures. The strongest negative correlations with seizure reduction were with 5-hydroxyvalproate, a metabolite of the antiepileptic drug valproate (VPA), and the two lipids palmitoyl-linoleoyl-glycerol (2), and 1-stearoyl-2-linoleoyl-GPI.

**Table 2:** Spearman correlation between metabolites and relative seizure reduction.

Metabolites with positive correlations to seizure reduction	r	CI (95%)	p	fdr
1-(1-enyl-palmitoyl)-2-linoleoyl-GPC^a^	0.85	0.64 to 0.98	0.0001	0.11
*Propionic acid*	*0.83*	*0.00* to *0.99*	*0.002*	*0.01*
3-Hydroxyisobutyrate	0.76	0.22 to 0.94	0.002	0.53
Thymol sulfate	0.76	0.36 to 0.92	0.002	0.53
Fibrinopeptide A (2-15)	0.72	0.28 to 0.93	0.004	0.53
Fibrinopeptide A (3-15)	0.71	0.28 to 0.90	0.004	0.53
*Isobutyric acid*	*0.70*	*−0.04 to 0.95*	*0.017*	*0.06*
3-Indoleglyoxylic acid	0.69	0.22 to 0.91	0.006	0.54
Vanillylmandelate	0.68	0.22 to 0.90	0.007	0.54
*Valeric acid*	*0.68*	*0.11* to *0.94*	*0.023*	*0.06*
Hydroquinone sulfate	0.66	−0.06 to 0.96	0.010	0.6
N-Acetylmethionine	0.64	0.09 to 0.90	0.014	0.64
Glucose	0.64	0.10 to 0.89	0.014	0.64
Ribonate	0.62	−0.06 to 0.92	0.019	0.67
1-(1-enyl-palmitoyl)-2-oleoyl-GPC^a^	0.61	0.16 to 0.86	0.021	0.67
1-(1-enyl-palmitoyl)-2-arachidonoyl-GPC^a^	0.61	−0.03 to 0.88	0.022	0.67
1-(1-enyl-palmitoyl)-2-arachidonoyl-GPE^a^	0.60	0.03 to 0.89	0.024	0.67
SUM^b^	0.80	0.47 to 0.94	0.001	NA

Metabolites with negative correlations to seizure reduction	r	CI (95%)	p	fdr
5-hydroxyvalproate	−0.75	−0.92 to −0.42	0.002	0.53
Palmitoyl-linoleoyl-glycerol (2)^a^	−0.71	−0.91 to −0.34	0.004	0.53
1-Stearoyl-2-linoleoyl-GPI	−0.71	−0.92 to −0.34	0.005	0.53
Ximenoylcarnitine^a^	−0.71	−0.92 to −0.20	0.005	0.53
N-Acetylhistidine	−0.69	−0.90 to −0.28	0.007	0.54
Linoleoyl-linoleoyl-glycerol (1)^a^	−0.68	−0.91 to −0.30	0.007	0.54
Linoleoyl-linoleoyl-glycerol (2)^a^	−0.67	−0.90 to −0.28	0.009	0.6
Palmitoyl-linoleoyl-glycerol (1)^a^	−0.66	−0.88 to −0.16	0.010	0.6
1-Palmitoyl-GPG^a^	−0.65	−0.87 to −0.29	0.011	0.64
1-Stearoyl-2-linoleoyl-GPE^a^	−0.64	−0.90 to −0.15	0.013	0.64
1-Stearoyl-2-oleoyl-GPE	−0.64	−0.91 to −0.10	0.013	0.64
Gamma-glutamylmethionine	−0.63	−0.87 to −0.08	0.015	0.67
Palmitoylcholine	−0.63	−0.94 to 0.05	0.016	0.67
1-Stearoyl-2-Docosahexaenoyl-GPE^a^	−0.62	−0.92 to −0.02	0.018	0.67
Gamma-glutamylalanine	−0.62	−0.89 to −0.10	0.018	0.67
1-stearoyl-GPG	−0.61	−0.90 to −0.09	0.021	0.67
Glycoursodeoxycholate	−0.6	−0.88 to −0.11	0.023	0.67

Results are ranked by correlation strength (r). A positive correlation coefficient r indicates that an increase in the metabolite correlates with increased therapeutic effect, i.e., relative seizure reduction, a negative r value indicates a negative relationship between metabolite change and seizure reduction. CI (95%) indicates the 95% confidence interval, p the p-value. Italics indicates SCFA which were analyzed in a separate, targeted assay.

a Indicates compounds that have not been officially confirmed by Metabolon based on a standard, but we are confident in their identity.

b Sum of individual changes of 1-(1-enyl-palmitoyl)-2-oleoyl-GPC, 1-(1-enyl-palmitoyl)-2-arachidonoyl-GPC and 1-(1-enyl-palmitoyl)-2-arachidonoyl-GPE.

Correlations of seizure reduction with changes in faecal microbe abundances were generally weaker, with only five species with p<0.05 (Table 3). Three belong to the genus Alistipes and an increase in all three, together with an increase in *Faecalibacterium prausnitzii,* correlated positively with seizure reduction, while only *Coprococcus catus* showed negative correlations. The correlation results of all microbial taxa with seizure reduction are provided in Supplementary Table S5.

**Table 3:** Spearman correlation between changes in microbial taxa and relative seizure reduction.

Microbes	r	CI (95%)	p	fdr
**Species with positive correlations to seizure reduction**				
*Alistipes shahii* WAL 8301	0.65	0.31 to 0.84	0.002	0.19
*Alistipes communis* 5CBH24	0.58	0.19 to 0.82	0.007	0.37
*Faecalibacterium prausnitzii* L2.6	0.46	−0.02 to 0.77	0.043	0.76
*Alistipes finegoldii* DSM 17242	0.45	−0.11 to 0.78	0.044	0.76
**Species with negative correlations to seizure reduction**				
*Coprococcus catus* GD.7	−0.47	−0.75 to 0.03	0.037	0.76

Results are ranked by correlation strength (r). A positive correlation coefficient r indicates that an increase in the taxon correlates with increased therapeutic effect, i.e., relative seizure reduction. A negative r value indicates a negative relationship between taxon increase and seizure reduction. CI (95%) indicates the 95% confidence interval, p the p-value.

**Fig. 3:**
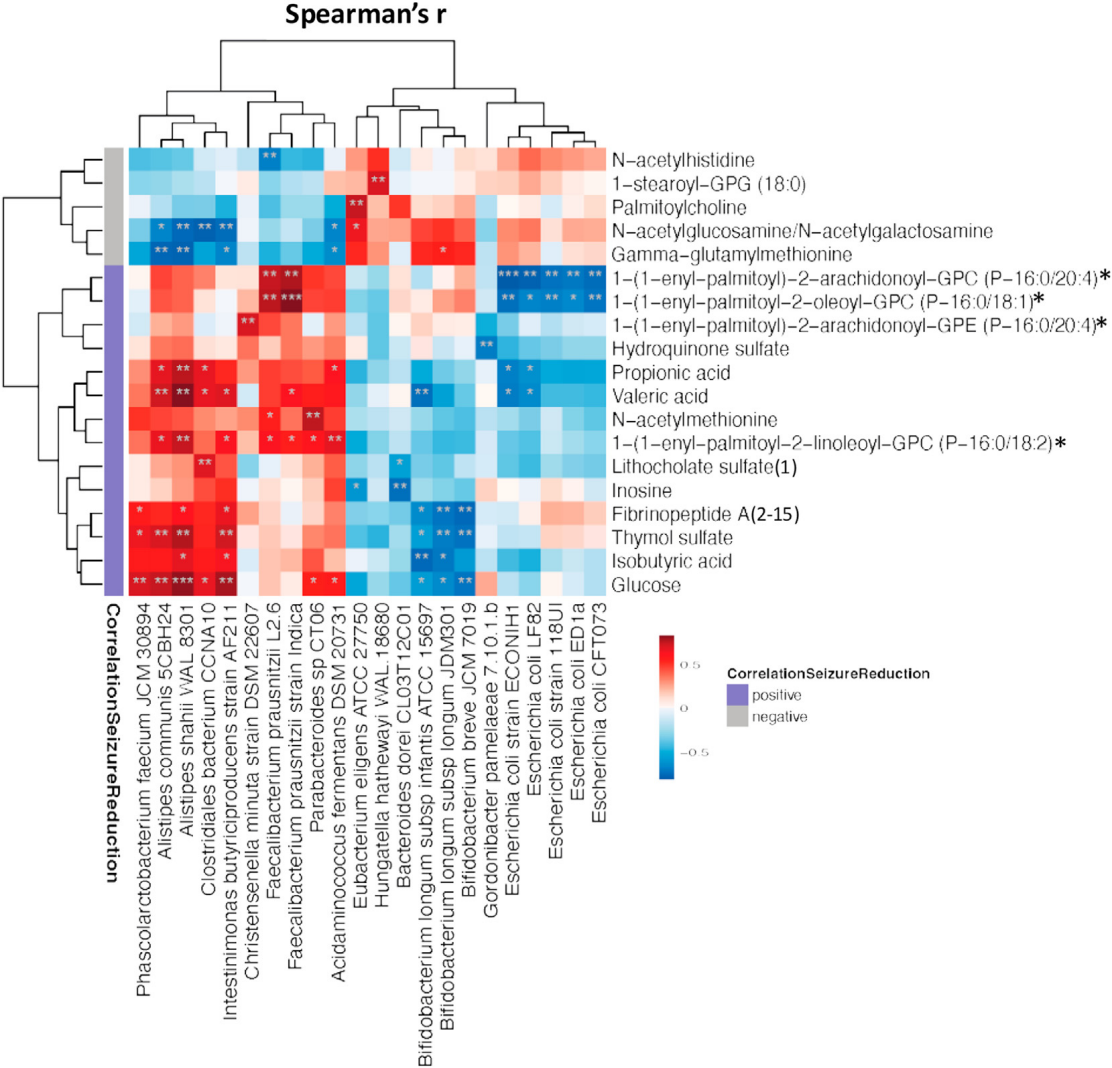
**Heatmap of Spearman correlation coefficients of microbial taxa with the most significant metabolites correlating with seizure reduction.** For clustering, Euclidean distances were used for rows and columns. Metabolite changes that positively correlated with seizure reduction are labelled in purple, while negative correlations are labelled in grey. Positive correlations are shown in red, negative correlations in blue. Taxa are only included in the heatmap if there was at least one correlation with a metabolite with p<0.01. ∗ indicates p<0.05, ∗∗ indicates p<0.01 and ∗∗∗ indicates p<0.001.

To investigate whether any of the most significant serum metabolite changes correlating with seizure reduction were associated with changes in the gut microbiota, we performed Spearman's correlation analysis between these metabolites and all 99 microbial species. All metabolites and all species with at least one significant correlation (p<0.01) are shown in Fig. 3. We identified two clusters of species, one with positive correlations with beneficial metabolite changes and one with negative correlations to beneficial metabolite changes. In the first cluster, the strongest positive correlations were found for two *F. prausnitzii* strains with two plasmalogens (1−(1−enyl−palmitoyl)−2−arachidonoyl−GPC, PC P-16:0/20:4; and 1−(1−enyl−palmitoyl)−2−oleoyl−GPC, PC P-16:0/18:1). The two Alistipes strains, *Alistipes shahii WAL 8301* and *Alistipes communis 5CBH24*, which had strongest positive correlations with seizure reduction, and *Intestinimonas butyriciproducens* correlated significantly with (1−(1−enyl−palmitoyl)−2−linoleoyl−GPC (PC P-16:0/18:2)) the plasmalogen with the strongest correlation to seizure reduction, as well as with thymol sulfate, glucose and the SCFAs propionate and valerate. These species also displayed the strongest negative correlations with changes in metabolites that were negatively associated with seizure reduction, (*e.g.,* gamma−glutamylmethionine and N−acetylglucosamine/N−acetylgalactosamine). The second cluster of species showed opposite associations, with three infant type Bifidobacteria negatively correlating with Fibrinopeptide A, thymol sulfate, isobutyrate and glucose. Five *Escherichia coli* strains negatively correlated with the two plasmalogens that were positively associated with *F. prausnitzii.*

## Discussion

Few clinical studies have investigated serum metabolomic changes during KD^7^, ^8^, ^9^ of which two involved patients with epilepsy.^8^^,^^9^ We here uncover links of these changes to gut microbiota and seizure response. In our cohort, KD induced multiple metabolite changes, including several lipid pathways as well as amino acids and vitamins.

Amongst the most significant metabolite changes correlating positively with seizure reduction, we identified four structurally related plasmalogens. Plasmalogens are a unique class of membrane glycerophospholipids that contain a fatty alcohol with a vinyl-ether bond at the *sn*-1 position, and a (poly)unsaturated fatty acid at the *sn*-2 position of the glycerol backbone.^18^ The head group is most frequently a choline (PlsCho; plasmenylcholines) or an ethanolamine (PlsEtn; plasmenylethalomines) and PlsEtn can be metabolised to PlsCho. In the human brain, plasmalogens are the predominant phospholipids in the gray and white matter of the frontal and parietal cortex.^18^ 58% of all glycerophosphoethanloamines are PlsEtn with 80% in the white matter of frontal, parietal, and temporal cortex as well as the cerebellum. The plasmalogen PE P-16:0/20:4, identified in our study, has been detected in all these brain regions. Plasmalogens influence membrane properties and membrane-associated signalling.^19^ They contribute to the organisation of neuronal membranes and the myelin sheath and low levels have been associated with several neurological diseases including Alzheimer's disease (AD), Parkinson's (PD) and autism spectrum disorder (ASD), a common co-morbidity of epilepsy.^19^ A role of the gut microbiota in these disorders has also been suggested.^20^ Plasmalogens have antioxidant properties^21^ and inhibit neuroinflammation,^22^ properties that may help counteract the damaging effects of seizures on brain tissue. Low levels of plasmalogens have been proposed to alter the biophysical properties of synaptic vesicles leading to disturbed neurotransmitter release^23^ which may influence seizure threshold. Genetic disorders disrupting peroxisome function (*e.g.,* Rhizomelic chondrodysplasia punctata (RCDP) and Zellweger spectrum disorder) are accompanied by plasmalogen deficiency and seizures.^24^^,^^25^

Although a specific transporter for plasmalogens across the blood–brain barrier has not been identified yet, oral supplementation with plasmalogen increased blood plasmalogen content in patients with AD and PD and improved cognitive function and/or mental conditions.^26^^,^^27^ Enhanced plasmalogen concentrations were detected in the mouse brain upon oral ingestion.^28^ Oral plasmalogens have anti-inflammatory and anti-amyloidogenic properties,^29^ can activate microglia and improve LPS-induced memory loss via increased gene expression of brain-derived neurotrophic factor (BDNF) in a mouse model of AD.^30^ In the hippocampus, oral ingestion of plasmalogens increased synaptic plasticity, neurogenesis and the number of dendritic spines,^28^^,^^31^ all processes potentially influencing seizure susceptibility.

Interestingly, the Canadian company Med-Life Discoveries LP has synthetic plasmalogen precursors in various clinical trials for the treatment of AD, PD, Multiple Sclerosis and RCDP.^32^

All four serum plasmalogens correlating positively with seizure reduction in our Swedish cohort were recently found increased in mouse faecal metabolites after receiving faecal transplants from patients with epilepsy in an independent American cohort after one month of KD compared to faecal transplants from the same patients before starting KD.^33^ These plasmalogens were indirectly linked to transcriptional changes in the mouse hippocampus, specifically in the Wnt/β-catenin pathway and Glycosylphosphatidylinositol (GPI) anchor biosynthetic process, pathways implicated in seizure susceptibility. Despite differences in the study population (American vs. Swedish), and length of KD treatment (one month vs. three) we identify the same four plasmalogens as important in seizure regulation using different analysis approaches. Thus, findings by Lum *et al*. support our main findings and our conclusion that plasmalogens might contribute to the anti-seizure effects of KD. The fact that the serum plasmalogens positively correlating with seizure reduction in our study were identified in the feces of the mice by Lum *et al*. strengthens our hypothesis that the gut microbiota plays a role in their metabolism. In addition, the microbes correlating with plasmalogens identified in our study all belong to bacterial orders containing plasmalogen encoding genes.^34^

Furthermore, propionate and thymol sulfate were identified as positively correlating with seizure reduction during KD treatment in our study. Propionate is one of the most abundant SCFA in the human body produced during gut microbial fermentation. Intragastrical supplementation at physiologically relevant levels has been shown to attenuate mitochondrial disruption, hippocampal apoptosis and neurological deficits in pentylenetetrazol (PTZ)-induced seizures in mice.^35^ Thymol sulfate is functionally related to thymol, a volatile phenol found in herbal plants including thyme, oregano, and basil. This dietary component has antioxidant, anti-inflammatory and anti-epileptic activities.^36^

In addition to those metabolites correlating positively with seizure reduction, we also noted several molecules with negative correlations to seizure reduction. For example, the levels of several diacylglycerols, either with free glycerol or conjugated to phosphatidylethanolamine (PE), evidenced negative correlations with seizure reduction. Many of these species contained the 16-carbon saturated fatty acid palmitic acid or were enriched in 18-carbon fatty acids including saturated (stearic acid), mono-unsaturated (oleic acid), and polyunsaturated (linoleic acid) fatty acids (*e.g.,* PE 18:0/18:1 and PE 18:0/18:2). These associations are likely linked with dietary intake given that palmitic acid is the primary saturated fatty acids in most diets, while linoleic is the most common dietary source of polyunsaturated fatty acids.

Two gamma-glutamyl amino acids negatively correlated with seizure reduction, of which one significantly negatively correlated with intestinal microbes. Olson *et al*. previously demonstrated that specific gut bacteria decreased serum levels of gamma-glutamyl amino acids which was associated with seizure reduction in mice.^12^ Our findings indicate that this mechanism may also contribute to lower seizure propensity in patients.

We identified four species positively correlating with seizure reduction and one negatively (r>|0.4|). Out of the four species, three belonged to the genus Alistipes frequently found in the gut microbiota with potential involvement in neurological diseases.^37^ The correlations were not as strong as for the metabolites which may indicate a less direct influence of the gut microbiota. However, it needs to be kept in mind that taxonomic annotation of faecal microbial DNA does not directly reflect bacterial activity or gene expression in the gastrointestinal tract while measuring circulatory metabolites is a more direct measure of metabolism.

We here identified one cluster of gut microbial species positively associated with beneficial serum metabolite changes and one with opposing associations. The cluster associated with beneficial metabolite changes comprises health-associated bacterial species such as *F. prausnitzii* and *I. butyriproducens* as well as bacteria with yet less well characterised roles for human health such as *Alistipes, Acidaminococcus fermentans* and *Phascolarctobacterium faecium*. The cluster with negative associations of metabolites on seizure reduction is dominated by five different *E. coli* strains and three infant-type Bifidobacteria. We have previously shown that these bifidobacterial strains were higher in responders before starting KD and a decrease in abundance of these was associated with decrease in systemic inflammation and anti-seizure response to KD.^14^ We have also shown an increase of *E. coli* in the gut microbiota during KD.^13^^,^^14^ An expansion of *E. coli* has been associated with several chronic intestinal diseases such as colorectal cancer^38^ and inflammatory bowel disease (IBD).^39^ We now identify negative associations of *E. coli* with plasmalogens, propionate and valerate, which were all positively linked to seizure reduction. Thus, we postulate that an increase in *E. coli* during KD may hamper seizure reduction.

### Strengths and limitations of the study

Our exploratory study is unique using extensive metagenomic and metabolomic profiling of patients with epilepsy before and during KD correlating individual changes to seizure reduction. However, due to the generally low number of patients starting KD treatment, we were limited to a small cohort with a large age range and heterogenous epilepsy phenotypes as well as typical individual variations in the gut microbiota composition and serum metabolite profiles. This led to limited power and relatively large 95% confidence intervals for correlation strengths of taxa and some metabolites to seizure reduction. High fdr values were a result of limited sample size and a high number of variables, i.e., 99 microbial taxa and 995 serum metabolites. However, individual variations and confounders such as age and treatment with different ASMs could be limited and statistical power was increased by applying a paired analysis and correlating individual changes in metabolites and microbes to seizure reduction for each patient.

Relative seizure reduction was calculated using seizure calendars made by parents and other caregivers who had long-term experience of monitoring the seizure frequency of their child. Before study start a careful review of each child's seizure type(s) was done. The accuracy of the monitoring of the individual child would likely be similar during the observation before and during treatment. Thus, the use of relative seizure reduction as the outcome measure could minimise systematic errors. Our approach used a subjective evaluation of seizure frequency but an objective measure as EEG monitoring is not suitable during month-long monitoring.

### Conclusions

We here present metabolomic changes observed in serum of patients with epilepsy undergoing KD treatment. While our cohort was small and heterogenous, we were able to correlate individual seizure reduction to individual changes in the gut microbiota community and serum metabolites, with plasmalogens showing the strongest associations. This is an exploratory study identifying metabolites and bacteria with potential effects on seizure thresholds in patients. Our findings need further validation in independent studies for their clinical importance. Anti-seizure strategies could then be developed to promote the increase of bacterial species and/or serum metabolites positively associated with seizure reduction, while limiting the expansion or decreasing the abundance of bacteria and/or metabolites negatively associated with therapeutic outcome. As one third of patients with epilepsy suffers from pharmaco-resistant seizures, new therapeutic approaches are desperately needed for improving quality of life for patients and caregivers as well as for decreasing the socioeconomic burden of epilepsy.

## Contributors

S.P.-N. conceived the idea and designed the experiments. M.D. acquired the ethical permissions and parental consent, was responsible for patient identification, sample collection, diet treatment, and patient data analyses. S.P.-N. performed the metabolomics and metagenomics data analyses. M. D. and S. P.-N. accessed and verified the data. M. D. and S. P.-N. wrote the manuscript. C.E.W. advised on metabolomics analyses, assisted in the interpretation of metabolomics results and in writing the manuscript. Funding was obtained by S.P.-N. and M.D. All authors have read and approved the manuscript.

## Data sharing statement

Raw sequencing data are available at the European Nucleotide Archive under study accession number PRJEB28847 and PRJEB49521. Normalised metabolomics data for each sample and each metabolite can be found in Supplementary Table S1.

## Declaration of interests

None of the authors has any conflict of interest to disclose. We confirm that we have read the Journal's position on issues involved in ethical publication and affirm that this report is consistent with those guidelines.
